# Analysis of factors associated with waiting times for GP appointments in Finnish health centres: a QUALICOPC study

**DOI:** 10.1186/s13104-018-3316-7

**Published:** 2018-04-03

**Authors:** Elina Tolvanen, Tuomas H. Koskela, Kari J. Mattila, Elise Kosunen

**Affiliations:** 1 Faculty of Medicine and Life Sciences, University of Tampere, Tampere, Finland; 2 Pirkkala Municipal Health Centre, Pirkkala, Finland; 3 Science Centre, Pirkanmaa Hospital District, Tampere, Finland; 4 Centre for General Practice, Pirkanmaa Hospital District, Tampere, Finland

**Keywords:** Access to health care, Waiting times, GP appointments at health centres, General practitioners, Finland, Primary health care

## Abstract

**Objective:**

Access to care is a multidimensional concept, considered as a structural aspect of health care quality; it reflects the functioning of a health care organization. The aim of this study was to investigate patients’ experiences of access to care and to analyse factors associated with waiting times to GP appointments at Finnish health centres. A questionnaire survey was addressed to Finnish GPs within the Quality and Costs of Primary Care in Europe study framework. Two to nine patients per GP completed the questionnaire, altogether 1196. Main outcome measures were waiting times for appointments with GPs and factors associated with waiting times. In addition, patients’ opinions of access to appointments were analysed.

**Results:**

Of the 988 patients who had made their appointment in advance, 84.9% considered it easy to secure an appointment, with 51.9% obtaining an appointment within 1 week. Age and reason for contact were the most significant factors affecting the waiting time. Elderly patients tended to have longer waiting times than younger ones, even when reporting illness as their reason for contact. Thus, waiting times for appointments tend to be prolonged in particular for the elderly and there is room for improvement in the future.

**Electronic supplementary material:**

The online version of this article (10.1186/s13104-018-3316-7) contains supplementary material, which is available to authorized users.

## Introduction

Access to care is considered one of the key elements of primary health care (PHC) [1, 2]. Better access to PHC services is associated with higher patient satisfaction and quality of care [3–5], even if some controversial results have been reported [6]. Access to care is a complex and multidimensional concept including aspects from affordability to availability [7, 8]. According to Donabedian’s framework, access to care is considered as a structural component of health care quality [9, 10]. Among different aspects of access to PHC, geographical access, the waiting time for a doctor’s appointment, the ease of contacting the clinic by phone and the clinic’s opening hours are considered important [1, 11–13].

In Finland, the PHC system is universal and taxation-based, mainly provided by municipality-arranged, multidisciplinary health care centres. Regulated by the Finnish Health Care Act, a patient’s need for non-emergency treatment in PHC must be evaluated by health care professionals within 3 days of initial contact [14]. This is usually made by nurses.

Access to health care centres in Finland have deteriorated in past decades, despite several government acts aiming to develop Finnish health care. In 2007, 72% of the Finnish study population estimated that an appointment could be obtained within 3 days [15]. In 2015, the percentage of patients reporting easy access to primary health care had decreased from 38 to 18% over the 15-year study period [16]. Official statistics [17] and recent telephone survey results [18] support these findings.

There is only limited information available about the variation of access in different patient groups or the factors associated with long waiting times. Previous studies have shown that patient’s age [4, 15, 19, 20] or working status [4, 15, 21] are associated with access to PHC services, but the findings are inconsistent among studies. Furthermore, having a chronic illness [15] and lower income [22, 23] have been associated with poorer accessibility. However, when regarding factors related to access to care, the access of a single patient is probably more dependent on how well the health care system is functioning than on the individual characteristics of the patient or the GP. This effect may be stronger in Finland, where GPs are employed mainly by public, municipal organisations compared to countries where GPs work mainly as independent practitioners.

The goal of the present study was to assess waiting times for GP appointments at health centres in Finland using the Quality and Costs of Primary Care in Europe (QUALICOPC) study data. We analysed factors associated with waiting times to GP appointments and studied patients’ experiences of access to care.

## Main text

### Materials and methods

We used the Finnish data collected for the international QUALICOPC study, which is aimed to evaluate PHC systems in 31 European countries along with Australia, Canada and New Zealand. The QUALICOPC study design and the international process of developing the study questionnaires are described elsewhere [11, 13]. In the study framework, there are questionnaires for GPs, their patients (PE = Patient Experience and PV = Patient Values) and fieldworkers “to evaluate the system, the practice and the patient” [11]. The original questionnaires were translated from English to Finnish with a formal forward-back translation process.

**Fig. 1:**
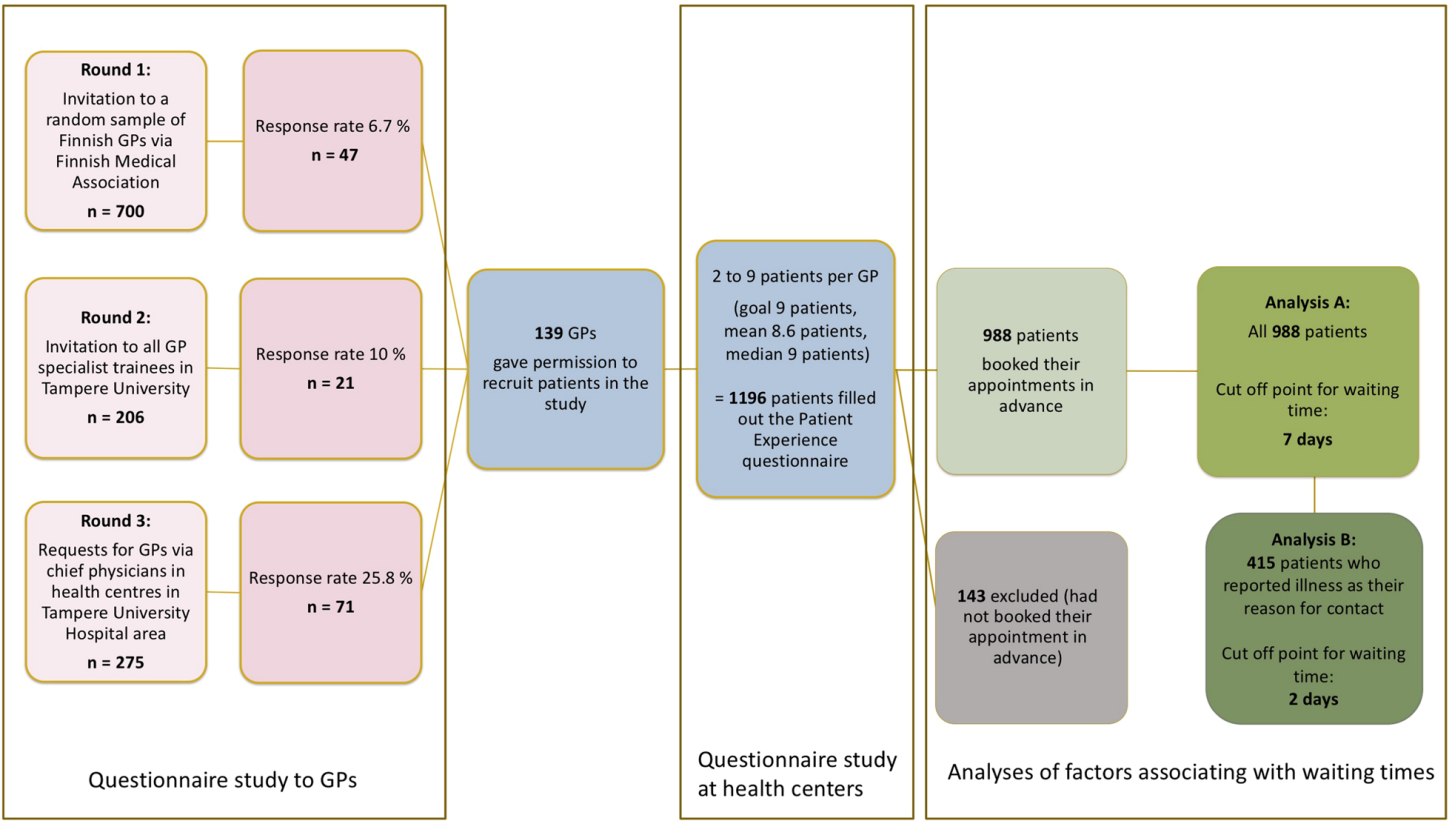
Gathering the study sample of Finnish GPs and their patients for the QUALICOPC study and design of the analyses considering waiting times

According to the QUALICOPC study design, the goal was to reach 220 GPs in each country and nine patients for each GP to fill out the Patient Experience questionnaire. The Finnish data were collected in 2012. The purpose was to get a random sample of Finnish GPs, but unfortunately the response rates were so low that completing recruitments were needed. The process of gathering the study sample of GPs is presented in Fig. 1.

Ultimately, a total of 139 GPs agreed to participate in the study according to the protocol. The patients were recruited by a trained fieldworker and asked to fill out the questionnaire at the health centre immediately after the appointment with the GP. Two to nine patients per GP were recruited, altogether 1196, with a median of nine patients and a mean of 8.6 patients.

All patients who filled out the PE questionnaire were included in the analyses considering geographical access and ease of contacting the clinic. When considering waiting times for GP appointments, patients who had not booked their appointment in advance (n = 143) were excluded; thus, 988 patients were included in these analyses. Furthermore, we separated the subgroup of patients who reported illness as the reason for contact (n = 415). The waiting time was asked by a question “how many days did you wait for the appointment” with answer alternatives of zero, one, two to seven or more than 7 days. The cut off points were 7 or 2 days, depending on the group. The setting is presented in Fig. 1.

All background factors were included as covariates in final models. For the main reason for GP appointments, different options in the QUALICOPC questionnaire were categorised as following: “illness”, “non-urgent check-up” (a medical check-up or second opinion), “need for a medical document” (to get a prescription and/or a referral and/or a medical certificate) and “other or several”.

In the statistical analysis (IBM SPSS, version 21), descriptive statistics, cross-tabulation and bivariate logistic regression analysis were used to examine the background factors and to find variables exerting the strongest effect on waiting times. Secondly, multivariable logistic regression models were created. Due to the collecting method, the data could be clustered, meaning that the waiting time of the patient could depend on which health centre or GP the patient was visiting. Thus, multi-level modelling, i.e. generalized linear mixed-effect models were fitted using function glmer in R Software environment for statistical computing and graphics, version 2.13.0. Random intercept was used to account variation in number of patients per GP.

### Results

A total of 1196 patients completed the QUALICOPC Patient Experience questionnaire. The distributions of the background factors are presented in Additional file 1. The mean age of the patients was 59 years (range 18–97 years), and 51.5% were over 65 years old.

The patients rated geographical access to care fairly positively (data not shown). The majority (82.8%) could reach a health care centre within 20 min and almost all (97.9%) within 40 min. Altogether, 91.4% felt that the practice was not too far away from their home or workplace. One fifth (22.2%, n = 266) of the patients reported having to wait too long on the telephone when calling the practice. Similarly, 20.0% (n = 239) felt that the opening hours of the practice were too limited.

Of the 988 patients who had booked their appointment in advance, the majority (84.9%, n = 894) agreed that it was easy to make an appointment. The waiting times for consultations distributed by background factors are presented in Table 1. Altogether, 51.9% of the patients reported a waiting time of 1 week or less. Of the 415 patients who reported illness as the reason for contact, 185 (44.6%) reported a waiting time of 2 days or less.

**Table 1:** Length of waiting times for an appointment by background factors

	Appointment in advance (n = 988)		Illness as reason for contact (n = 415)	
	Within 7 days, n (%)	More than 7 days, n (%)	Within 2 days, n (%)	More than 2 days, n (%)
Age (years)				
< 30	57 (11.3)	22 (4.7)	20 (10.8)	15 (6.6)
30–49	108 (21.4)	74 (15.7)	53 (28.6)	42 (18.6)
50–69	200 (39.6)	191 (40.6)	78 (42.2)	76 (33.6)
≥ 70	140 (27.7)	183 (38.9)	34 (18.4)	93 (41.2)
Sex				
Male	175 (34.2)	172 (36.3)	62 (33.5)	69 (30.0)
Female	337 (65.8)	302 (63.7)	123 (66.5)	161 (70.0)
Type of residence				
Urban	234 (46.2)	225 (48.2)	81 (44.8)	106 (46.7)
Rural	273 (53.8)	242 (51.8)	100 (55.2)	121 (53.3)
Working status				
Working	152 (29.7)	88 (18.6)	76 (41.3)	37 (16.1)
Retired	261 (51.1)	311 (65.8)	76 (41.3)	147 (63.9)
Other or several	98 (19.2)	74 (15.6)	32 (17.4)	46 (20)
Education				
Lower-level	314 (62.1)	314 (67.1)	109 (59.6)	157 (68.9)
Middle-level	145 (28.7)	111 (23.7)	52 (28.4)	49 (21.5)
Higher-level	47 (9.3)	43 (9.2)	22 (12.0)	22 (9.6)
Income (own estimate)				
Below average	187 (36.7)	195 (41.3)	61 (33.2)	109 (47.8)
Around average	283 (55.6)	247 (52.3)	109 (59.2)	109 (47.8)
Above average	39 (7.7)	30 (6.4)	14 (7.6)	10 (4.4)
Chronic disease^a^				
No	178 (35.0)	120 (25.4)	68 (37.0)	62 (27.3)
Yes	331 (65.0)	353 (74.6)	116 (63.0)	165 (72.7)
Health status (own estimate)				
Very good/good	212 (41.5)	178 (37.6)	78 (42.6)	69 (30.0)
Fair/poor	299 (58.5)	296 (62.4)	105 (57.4)	161 (70.0)
Has an assigned GP				
No	156 (30.6)	148 (31.7)	62 (33.5)	76 (33.3)
Yes	354 (69.4)	319 (68.3)	123 (66.5)	152 (66.7)
Reason for appointment				
Illness	284 (55.6)	131 (27.6)	185 (44.6)	230 (55.4)
Non-urgent check-up	71 (13.9)	162 (34.1)		
Need for a medical document	53 (10.4)	88 (18.5)		
Other or several	103 (20.2)	94 (19.8)		

^a^Having a long-standing disease or condition such as diabetes, high blood pressure etc

The results of bivariate analyses of all patients who had made their appointment in advance as well as the subgroup of patients who reported an illness as their reason for contact are presented in Additional file 2. In the bivariate analyses, it appeared that younger age, more urgent reason for contact, more active working status, higher income and absence of a chronic disease had associations on shorter waiting times.

The results of the multivariable analyses of both groups are presented in Table 2. In multivariable analyses, patients reporting an illness obtained their appointments evidently faster than patients with other reasons (e.g. OR for the non-urgent check-up group was 4.6 (95% CI 3.2–6.5, *p* < 0.001). Elderly patients had longer waiting times more often than the younger (OR 1.02 per year, 95% CI 1.003–1.03, *p* < 0.001), even if reporting an illness as the reason for contact. In addition, in cases of illness, actively working patients succeeded to have shorter waiting times (OR for retired patients was 2.4 (95% CI 1.2–4.7, *p* < 0.001). The interpretation of the results did not change after taking into account the clustered nature of the data by multi-level modelling.

**Table 2:** Results of multivariable analyses for (A) patients who booked their appointments in advance (n = 988) and (B) the subgroup of patients who reported illness as their reason for contact (n = 415)

Category (RC = reference)	(A) Patients who booked their appointment in advance (n = 988)				(B) Patients who reported illness as their reason for contact (n = 415)			
	n	Odds ratio (OR)	95% CI for OR	*p* value	n	Odds ratio (OR)	95% CI for OR	*p* value
Age								
	975	*1.02*	*1.003–1.03*	0.02	411	*1.02*	*1.001–1.04*	0.04
Data missing/NA	13				4			
Sex								
Male (RC)	347	1.0			131	1.0		
Female	639	1.0	0.8–1.4	0.81	284	1.1	0.7–1.8	0.72
Data missing/NA	2				0			
Type of residence								
Urban (RC)	459	1.0			187	1.0		
Rural	515	0.9	0.7–1.2	0.55	221	1.0	1.6–1.5	0.96
Data missing/NA	14				7			
Working status								
Working (RC)	240	1.0			113	1.0		
Retired	172	1.3	0.8–2.0	0.28	223	*2.4*	*1.2–4.7*	0.01
Other or several	572	1.3	0.8–2.1	0.27	78	2.2	1.0–4.7	0.04
Data missing/NA	4				1			
Education								
Lower-level (RC)	628	1.0			266	1.0		
Middle-level	256	1.0	0.7–1.4	0.96	101	1.0	0.6–1.8	0.90
Upper-level	90	1.5	0.9–2.6	0.11	44	1.4	0.6–3.0	0.38
Data missing/NA	14				4			
Income (own estimate)								
Below average (RC)	382	1.0			170	1.0		
Around average	530	0.8	0.6–1.1	0.18	218	0.8	0.5–1.2	0.16
Above average	69	0.6	0.3–1.2	0.14	24	0.5	0.2–1.4	0.20
Data missing/NA	7				3			
Chronic disease^a^								
No (RC)	298	1.0			130	1.0		
Yes	694	1.2	0.9–1.7	0.21	281	1.0	0.6–1.7	0.92
Data missing/NA	9				4			
Health status (own estimate)								
Very good/good (RC)	390	1.0			147	1.0		
Fair/poor	595	0.9	0.7–1.3	0.60	266	1.0	0.6–1.6	0.91
Data missing/NA	9				2			
Has an assigned GP								
No (RC)	304	1.0			138	1.0		
Yes	673	0.8	0.6–1.0	0.10	275	0.9	0.6–1.5	0.72
Data missing/NA	11				2			
Reason for appointment								
Illness (RC)	415	1.0						
Non-urgent check-up	233	*4.5*	*3.2–6.5*	< 0.001				
Need for a medical document	141	*4.0*	*2.6–6.2*	< 0.001				
Other or several	197	*2.0*	*1.4–2.9*	< 0.001				
Data missing/NA	2							

Waiting time more than 7 days (A) or more than 2 days (B) as a dependent variable

*CI* confidence interval, *NA* non-applicable

Italic values indicate significance of p value (*p* < 0.05)

^a^Having a long-standing disease or condition such as diabetes, high blood pressure, etc

### Discussion

According to the results of the present questionnaire survey, approximately half of the patients had obtained an appointment with a GP within a week. Younger age and more urgent reason for contact were the most significant factors associated with faster access to GP appointments. In the subgroup who reported illness as the reason for seeking an appointment, younger age and active working status were associated with shorter waiting times.

In our study, patients with non-urgent matters waited longer than those with illness, which seems quite reasonable. However, in cases of illness, younger and actively working patients obtained their appointments more quickly. In the broad context of access to care, the waiting time for an appointment may reflect not only the individual characteristics of the patient but also how the health care organisation functions. Thus, according to this study, our health care system seems to favour younger and working people in terms of access to care.

The majority of respondents evaluated access to GP appointments positively either in terms of securing an appointment or contacting the clinic by telephone. For the sake of comparison, in a survey for Finnish Medical Association, 27% of respondents reported some or major problems regarding waiting times for GP appointments in health centres [18]. On the other hand, in a Finnish study conducted 20 years ago, 44% of patients having a non-acute problem had to wait more than a week for an appointment, while 19% waited over 2 weeks [24]. In Finland, people seem to accept longer waiting times for non-urgent matters [25]. This should be taken into consideration when comparing these results with those from other countries.

In the QUALICOPC study, the response rate among GPs varied a lot between countries. The goal of 220 GPs was not reached in all the countries [26], which occurred also in this study. Nevertheless, there are several strengths with the current sample. It includes a large number of patients from both urban and rural areas, the patients represent different age groups and the age distribution correlates well with the national register profile of all the patients who used Finnish health centres in year 2013 [27]. Furthermore, the results regarding waiting time lengths in this study are in line with the nationally registered information [17]. We therefore regard that the sample represents the overall situation in Finland fairly well. In addition, the questionnaires were completed thoroughly and there were few missing responses, suggesting good quality data.

According to the design of the QUALICOPC study framework, the participating patients where those who had eventually obtained an GP appointment. Thus, with this kind of setting, the whole complexity of access to care could not be reached. However, with multivariable and multi-level analyses we were able to take several patient and organisational features into consideration, which is a strength of this study.

### Conclusions

In the present study, older age was associated with longer waiting times for GP appointments in Finnish health care centres. The results suggest that somehow our health care system favours younger and working patients. Improving access to care, especially in terms of equality, has to be one of the main goals in the future health care reforms.

## Limitations

- The study sample was not a random sample of Finnish population or patients but it was, according the international QUALICOPC framework, based on the patients of GPs who voluntarily participated in this study.
- Due to the completing data collection methods needed, the geographical representativeness may have suffered with emphasis placed on the situation in western Finland.
- The waiting time and reason for contact were based on patient's own reporting, not an objective evaluation or measurement.
- The opinion about the waiting times were not included in the QUALICOPC questionnaire, so that aspect could not be taken into account in this study.

## Additional files

**Additional file 1.** The description of the participants. This file contains a table considering the background factors of the current sample of patients.

**Additional file 2.** Results of bivariate analyses. This file contains a table considering bivariate analyses. In the analyses, we have used waiting time more than 7 days or more than 2 days as a dependent variable.

## References

[1] Kringos DS, Boerma WG, Hutchinson A, Van Der Zee J, Groenewegen PP (2010). The breadth of primary care: a systematic literature review of its core dimensions. BMC Health Serv Res.

[2] Kringos DS, Boerma WG, Bourgueil Y, Cartier T, Hasvold T, Hutchinson A, Lember M, Oleszczyk M, Pavlic DR, Svab I, Tedeschi P, Wilson A, Windak A, Dedeu T, Wilm S (2010). The european primary care monitor: structure, process and outcome indicators. BMC Fam Pract.

[3] Beaulieu MD, Haggerty J, Tousignant P, Barnsley J, Hogg W, Geneau R, Hudon É, Duplain R, Denis JL, Bonin L, Del Grande C, Dragieva N (2013). Characteristics of primary care practices associated with high quality of care. CMAJ.

[4] Kontopantelis E, Roland M, Reeves D (2010). Patient experience of access to primary care: identification of predictors in a national patient survey. BMC Fam Pract.

[5] Llanwarne NR, Abel GA, Elliott MN, Paddison CAM, Lyratzopoulos G, Campbell JL, Roland M (2013). Relationship between clinical quality and patient experience: analysis of data from the English quality and outcomes framework and the national GP patient survey. Ann Fam Med.

[6] Baker R, Bankart MJ, Murtagh GM (2009). Do the quality and outcomes framework patient experience indicators reward practices that offer improved access?. Br J Gen Pract.

[7] Gulliford M, Figueroa-Munoz J, Morgan M, Hughes D, Gibson B, Beech R, Hudson M (2002). What does ‘access to health care’ mean?. J Health Serv Res Policy.

[8] Levesque JF, Harris MF, Russell G (2013). Patient-centred access to health care: conceptualising access at the interface of health systems and populations. Int J Equity Health.

[9] Donabedian A (1988). The quality of care: how can it be assessed?. JAMA J Am Med Assoc.

[10] Donabedian A (1966). Evaluating the quality of medical care. Milbank Mem Fund Q.

[11] Schäfer WL, Boerma WG, Kringos DS, De Maeseneer J, Gress S, Heinemann S, Rotar-Pavlic D, Seghieri C, Svab I, Van den Berg MJ, Vainieri M, Westert GP, Willems S, Groenewegen PP (2011). QUALICOPC, a multi-country study evaluating quality, costs and equity in primary care. BMC Fam Pract.

[12] Haggerty JL, Lévesque JF, Santor DA, Burge F, Beaulieu C, Bouharaoui F, Beaulieu MD, Pineault R, Gass D (2011). Accessibility from the patient perspective: comparison of primary healthcare evaluation instruments. Healthc Policy.

[13] Schäfer WLA, Boerma WGW, Kringos DS, De Ryck E, Greß S, Heinemann S, Murante AM, Rotar-Pavlic D, Schellevis FG, Seghieri C, Van Den Berg MJ, Westert GP, Willems S, Groenewegen PP (2013). Measures of quality, costs and equity in primary health care instruments developed to analyse and compare primary care in 35 countries. Qual Prim Care.

[14] Terveydenhuoltolaki 30.12.2010/1326. Finnish health care act 30.12.2010/1326. http://www.finlex.fi/en/laki/kaannokset/2010/en20101326. Accessed 1 Nov 2014.

[15] Mäntyselkä P, Halonen P, Vehviläinen A, Takala J, Kumpusalo E (2007). Access to and continuity of primary medical care of different providers as perceived by the Finnish population. Scand J Prim Health Care.

[16] Raivio R, Jaaskelainen J, Holmberg-Marttila D, Mattila KJ (2014). Decreasing trends in patient satisfaction, accessibility and continuity of care in Finnish primary health care - a 14-year follow-up questionnaire study. BMC Fam Pract.

[17] Terveyden ja hyvinvoinnin laitos (THL). Hoitoonpääsy perusterveydenhuollossa 2013. http://www.thl.fi/fi/tilastot/tiedonkeruut/hoitoonpaasy-perusterveydenhuollossa. Accessed 1 Nov 2014.

[18] Suomen Lääkäriliitto - uutiset. Finnish Medical Association—news. http://www.laakariliitto.fi/uutiset/ajankohtaista/pitkat-jonot-terveyskeskuksiin-edelleen-ongelmana/. Accessed 12 Mar 2015.

[19] Ferro A, Kristiansson PMD (2011). Ecology of medical care in a publicly funded health care system: a registry study in Sweden. Scand J Prim Health Care.

[20] Paddison C, Elliott M, Parker R, Staetsky L, Lyratzopoulos G, Campbell JL, Roland M (2012). Should measures of patient experience in primary care be adjusted for case mix? Evidence from the English General Practice Patient Survey. BMJ Qual Saf.

[21] Virtanen P, Kivimäki M, Vahtera J, Koskenvuo M (2006). Employment status and differences in the one-year coverage of physician visits: different needs or unequal access to services?. BMC Health Serv Res.

[22] Van Doorslaer E, Wagstaff A, Van Der Burg H, Christiansen T, De Graeve D, Duchesne I (2000). Equity in the delivery of health care in Europe and the US. J Health Econ.

[23] Van Doorslaer E, Masseria C, Koolman X (2006). Inequalities in access to medical care by income in developed countries. CMAJ.

[24] Lember M, Kosunen E, Boerma W (1998). Task profiles of district doctors in Estonia and general practitioners in Finland. Scand J Prim Health Care.

[25] Papp R, Borbas I, Dobos E, Bredehorst M, Jaruseviciene L, Vehko T, Balogh S (2014). Perceptions of quality in primary health care: perspectives of patients and professionals based on focus group discussions. BMC Fam Pract.

[26] Groenewegen PP, Greß S, Schäfer W: General Practitioners’ Participation in a Large, Multicountry Combined General Practitioner-Patient Survey: Recruitment Procedures and Participation Rate. Int J of Fam Med. 2016;4929432. 10.1155/2016/4929432. 10.1155/2016/4929432 PMC4800081 27047689

[27] Terveyden ja hyvinvoinnin laitos, (THL). Perusterveydenhuolto 2013; Primärvård 2013; Primary health care services 2013. Accessed ISSN: 1798-0887; Tilastoraportti - Statistikrapport - Statistical report. Available at: http://urn.fi/URN:NBN:fi-fe2014111846346

