# Supplementary material for: Analysis of factors associated with waiting times for GP appointments in Finnish health centres: a QUALICOPC study
Source: BMC Res Notes. 2018 Apr 3;11:220. doi: 10.1186/s13104-018-3316-7 (PMC5883288; doi:10.1186/s13104-018-3316-7)
Supplement: Supplementary file 1 — Additional file 1. The description of the participants. This file contains a table considering the background factors of the current sample of patients. [file 13104_2018_3316_MOESM1_ESM.docx]

| **Additional file 1. Description of the participants (n = 1196).** | | **n** | | **%** | |  |
| --- | --- | --- | --- | --- | --- | --- |
| **Sex** |  |  | |  | |  |
|  | Male | 430 | | 36.0 | |  |
|  | Female | 761 | | 63.6 | |  |
|  | Data missing | 5 | | 0.4 | |  |
| **Age** |  |  | |  | |  |
|  | 18 - 29 years | 95 | | 8.0 | |  |
|  | 30 – 49 years | 227 | | 19.0 | |  |
|  | 50 – 69 years | 467 | | 39.0 | |  |
|  | ≥70 years | 389 | | 32.5 | |  |
|  | Data missing | 18 | | 1.5 | |  |
| **Type of residence** | |  |  | |  | |
|  | Urban (big inner city, small town or suburbs) | 565 | | 47.2 | |  |
|  | Rural (mixed urban – rural or rural) | 615 | | 51.4 | |  |
|  | Data missing | 16 | | 1.3 | |  |
| **Education** | |  |  | |  | |
|  | No qualifications obtained/Pre-primary/Primary education (lower-level) | 757 | | 63.3 | |  |
|  | Upper secondary level of education (middle-level) | 313 | | 31.3 | |  |
|  | Post-secondary, non-tertiary education or higher (higher-level) | 105 | | 8.8 | |  |
|  | Data missing | 21 | | 1.8 | |  |
| **Working status** | |  |  | |  | |
|  | Employed, self-employed or in civil service | 291 | | 24.3 | |  |
|  | Retired | 679 | | 56.8 | |  |
|  | Student, unemployed, unable to work, mainly homemaker or combination of options | 218 | | 18.2 | |  |
|  | Data missing | 8 | | 0.7 | |  |
| **Income (own estimate)** | |  |  | |  | |
|  | Below average | 477 | | 39.9 | |  |
|  | Around average | 625 | | 52.3 | |  |
|  | Above average | 81 | | 6.8 | |  |
|  | Data missing | 13 | | 1.1 | |  |
| **Chronic disease*** | |  |  | |  | |
|  | Yes | 836 | | 69.9 | |  |
|  | No | 354 | | 29.6 | |  |
|  | Data missing | 6 | | 0.5 | |  |
| **Has an assigned GP** | |  |  | |  | |
|  | Yes | 810 | | 67.7 | |  |
|  | No | 372 | | 31.1 | |  |
|  | Data missing | 14 | | 1.2 | |  |
| **Booking of the appointment** | |  |  | |  | |
|  | In advance | 988 | | 82.6 | |  |
|  | No booking in advance/Data missing/Non-applicable | 208 | | 17.4 | |  |
| **Waiting time to the appointment, n = 988** | |  |  | |  | |
|  | 1–2 days | 269 | | 27.2 | |  |
|  | 2–7 days | 244 | | 24.7 | |  |
|  | More than a week | 475 | | 48.1 | |  |
| * = Having a long-standing disease or condition, such as diabetes, high blood pressure, etc. | | | | | |  |
