# Supplementary material for: Analysis of factors associated with waiting times for GP appointments in Finnish health centres: a QUALICOPC study
Source: BMC Res Notes. 2018 Apr 3;11:220. doi: 10.1186/s13104-018-3316-7 (PMC5883288; doi:10.1186/s13104-018-3316-7)
Supplement: Supplementary file 2 — Additional file 2. Results of bivariate analyses. This file contains a table considering bivariate analyses. In the analyses, we have used waiting time more than 7 days or more than 2 days as a dependent variable. [file 13104_2018_3316_MOESM2_ESM.docx]

**Additional file 2. Results of bivariate analyses for A) patients who booked their appointments in advance (n = 988) and B) the subgroup of patients who reported illness as their reason for contact (n = 415)**

**Waiting time more than seven days (A) or more than two days (B) as a dependent variable.**

|  |  | 1. **Patients who booked their appointment in advance (n = 988)** | | | 1. **Patients who reported illness as their reason for contact (N = 415)** | | |
| --- | --- | --- | --- | --- | --- | --- | --- |
| **Variable** | **Reference category (RC)** | **n** | **Odds ratio (OR)** | **95% CI for OR** | **n** | **Odds ratio (OR)** | **95% CI for OR** |
| **Age** | | | | | | | |
|  |  | 975 | *1.02* | *1.01–1.03* | 411 | *1.03* | *1.02–1.04* |
|  | Data missing/Non-applicable | 13 |  |  | 4 |  |  |
| **Sex** | | | | | | | |
|  | Male (RC) | 347 | 1.0 |  | 131 | 1.0 |  |
|  | Female | 639 | 0.9 | 0.7–1.2 | 284 | 1.2 | 0.8–1.8 |
|  | Data missing/NA | 2 |  |  | 0 |  |  |
| **Type of residence** | | | | | | | |
|  | Urban (RC) | 459 | 1.0 |  | 187 | 1.0 |  |
|  | Rural | 515 | 0.9 | 0.7–1.2 | 221 | 0.9 | 0.6–1.4 |
|  | Data missing/NA | 14 |  |  | 7 |  |  |
| **Working status** | | | | | | | |
|  | Working (RC) | 240 | 1.0 |  | 113 | 1.0 |  |
|  | Retired | 172 | *2.1* | *1.5–2.8* | 223 | *4.0* | *2.5–6.4* |
|  | Other or several | 572 | 1.0 | 0.8–1.9 | 78 | *3.0* | *1.6–5.4* |
|  | Data missing/NA | 4 |  |  | 1 |  |  |
| **Education** | | | | | | | |
|  | Lower-level (RC) | 628 | 1.0 |  | 266 | 1.0 |  |
|  | Middle-level | 256 | 0.8 | 0.6–1.0 | 101 | 0.7 | 0.4–1.0 |
|  | Upper-level | 90 | 0.9 | 0.6–1.4 | 44 | 0.7 | 0.4–1.3 |
|  | Data missing/NA | 14 |  |  | 4 |  |  |
| **Income (own estimate)** | | | | | | | |
|  | Below average (RC) | 382 | 1.0 |  | 170 | 1.0 |  |
|  | Around average | 530 | 0.8 | 0.6–1.1 | 218 | *0.6* | *0.3–0.8* |
|  | Above average | 69 | 0.7 | 0.4–1.2 | 24 | *0.4* | *0.2–0.95* |
|  | Data missing/NA | 7 |  |  | 3 |  |  |
| **Chronic disease ^a^** | | | | | | | |
|  | No (RC) | 298 | 1.0 |  | 130 | 1.0 |  |
|  | Yes | 694 | *1.6* | *1.2–2.1* | 281 | *1.6* | *1.03–2.4* |
|  | Data missing/NA | 9 |  |  | 4 |  |  |
| **Health status (own estimate)** | | | | | | | |
|  | Very good/good (RC) | 390 | 1.0 |  | 147 | 1.0 |  |
|  | Fair/Poor | 595 | 1.2 | 0.9–1.5 | 266 | *1.7* | *1.2–2.6* |
|  | Data missing/NA | 9 |  |  | 2 |  |  |
| **Has an assigned GP** | | | | | | | |
|  | No (RC) | 304 | 1.0 |  | 138 | 1.0 |  |
|  | Yes | 673 | 1.0 | 0.7–1.2 | 275 | 1.0 | 0.7–1.5 |
|  | Data missing/NA | 11 |  |  | 2 |  |  |
| **Reason for appointment** | | | | | | | |
|  | Illness (RC) | 415 | 1.0 |  |  |  |  |
|  | Non-urgent check-up | 233 | *4.9* | *3.5–7.0* |  |  |  |
|  | Need for a medical document | 141 | *3.6* | *2.4–5.4* |  |  |  |
|  | Other or several | 197 | *2.0* | *1.4–2.8* |  |  |  |
|  | Data missing/NA | 2 |  |  |  |  |  |
| *CI* Confidence interval, *NA* Non-applicable  Italic values indicate significance of p value (p < 0.05)  ^a^ = Having a long-standing disease or condition such as diabetes, high blood pressure, etc. | | | | | | | |
